# The Trans Man and the Warrior Women

**DOI:** 10.1089/trgh.2019.0047

**Published:** 2019-10-25

**Authors:** Kyan Lynch

**Affiliations:** Department of Obstetrics and Gynecology, University of Rochester Medical Center, Rochester, New York.

A transgender man, a doctor, a bioethicist, and an educator walk into a Women's Health Practice.

This isn't the setup to a joke—it's how I start each day.

I am a transgender man with a medical degree and a master's degree in medical humanities, compassionate care, and bioethics serving as the obstetrics and gynecology department education specialist at an academic medical center.

To say that my job is interesting would be a profound understatement. I am constantly navigating the complex waters of gender in medicine. These waters run through all sectors of the health care field, but their presence often goes unrecognized or unspoken. Not so in obstetrics and gynecology. Indeed, at my institution, as at many others, “Women's Health” is written on the door!

I knew, upon signing my contract, that this job would be interesting. That much was a given.

Far less clear, however, was whether I would *like* it. After all, I firmly believe that the concept of the gender binary is fraught, if not fraudulent. The idea that there are only two types of human, and only two ways to be human, has thoroughly, repeatedly, and often violently been debunked throughout history. The notion that humans come in only two flavors, male and female, and that these flavors are determined at birth and are fixed for the course of one's life is not only undermined by a growing body of medical evidence, but it is also in direct contradiction to my own lived experience.

I was born a girl. I know myself to be a man. End of story.

So, as I sat with my pen hovering above my unsigned contract, I had to ask myself, “Can I work in a department that exists to serve one half of the gender binary?”

The answer was not simple.

I am a gender rebel. I think that the button on our ultrasound machine that produces an “It's a boy!” label when a penis is spotted belongs in a dystopian novel. I abhor gender reveal parties. I am, in some ways, opposed to the very notion of a “Women's Health Practice.” Contract in hand, I was considering working at one.

Clearly, I signed the contract. But I did so with a rebellious flourish. I decided that I would take the position and use my role as an educator to change the department from the inside out. After all, words written on doors can be changed.

I committed myself to challenging the binary at every turn. I would change each “woman” to “patient,” each mention of “breastfeeding” to “lactation.” I would put a pronoun pin on each employee badge. I had a seat at the table, and I would use it.

Time passed. I settled in. I made good on many of the promises I had made to myself. I provided pronoun pins to my colleagues, edited our patient educational materials, and centered transgender issues at meetings. But something else happened too. Something unexpected.

I started to watch my female colleagues at work.

Despite my binary bashing, I must admit: these women who care for women are in a league of their own.

These women wake up to news that their rights are under attack. They receive threatening messages warning them that the care they give could get them jailed or killed. They hug their kids, their partners, their aging parents, and they walk out the door anyway.

They work with an awareness of their bodies at once intimate and detached: the fullness of the breast, the pull of the ligament, and the pregnant belly straining their scrubs. They haul their breast pumps up the hospital stairs and let the inhuman hum serve as the backdrop to meetings and lectures. There is no effort to hide or ignore the truths their bodies make obvious.

They knock on the door and enter smiling. They hold hands and read results and calm nerves and allay fears and break hearts and share joy and ease pain and shatter worlds and cry and comfort and soothe and repair and coax and inject and order and suture and probe and apologize and deliver and question and give and give and give and give.

They are givers of life and givers of self and they never stop. The world is always taking: reducing their reimbursements, devaluing their health, shortening their visits, decreasing their pay, cutting their resources, controlling their bodies, and criminalizing their care. They keep on giving anyway.

They are women and they care for women. If I am a gender rebel, they are gender warriors.

Watching these warrior women, I often catch myself in wistful reminiscence, recalling those moments when *I* was a gender warrior; the woman who didn't fit in and, therefore, stood out.

I consider the hurts and hallmarks of girlhood that I share with these women. My first period. My first slow dance. The first time I felt a man's gaze linger on my body, unwanted and unexpected. The insidious strengthening of that voice in my head telling me I shouldn't speak too loudly or want too much. The approving looks from the mothers in the stands as I turned a double play as the only girl on the little league team. The inexpressible joy of being my mother's daughter when she picked me up from school in scrubs.

I find myself recalling these moments and feel a swell of sweet nostalgia.

But then I look down at my flat chest. The one I paid for, bled for. The one I couldn't live without. The one I wouldn't be me without.

And that's when I know. I don't just have an interesting job, and I don't just like my job.

I love my job.

There is simply no better place for a gender rebel than a department full of gender warriors.

The women in this department are breaking gender rules constantly. But instead of justifying their breaches of norms by acting like men—as is a frequent impulse in academic medicine—they remain unapologetically, unashamedly, unmistakably women in all that they do.

I can think of no better role model for a transgender individual, particularly one looking to tackle the “cistem” of health care.

These women have helped me realize that my initial approach was internally inconsistent. When I took this job, I sought to remove gender from health care in the service of inclusivity, while at the same time preaching the importance of honoring a patient's gender identity.

Not only is this an untenable contradiction, it is also counterproductive to my cause. Erasing gendered language from our work doesn't remove gender bias from medicine. Quite the opposite! Removing gendered language simply allows the gender binary to continue, unchecked.

Instead, I need to take a page out of the gender warrior's book. I need to expand, not limit, the gender possibilities.

I'll start with the man in the mirror.

When I first started my transition, I was acutely aware of the messages I was conveying, through words, gestures, body language, tone, gait, and actions. I wanted to eschew the feminine in favor of the masculine. In short, I wanted to pass.

With the aid of testosterone, top surgery, and a brand-new wardrobe, more often than not, I am able to meet my goal.

However, now that I am surrounded by these warrior women, I feel the need to reach for more. I am not a cisgender man. I never will be. But these women have helped me to see what a glorious alternative it is to be a transgender man. I don't need to eschew the feminine or my reminiscences from girlhood. I can embrace them, learn from them, and derive strength from them. And when the need arises, I can speak the words of womanhood and be heard as a man.

Similarly, I don't need to strip our medical care of all references to gender. I need to create space for people of all genders and no gender to provide and receive care—without apologies, limitations, or excuses. Then, I need to celebrate it.

It's time, in other words, for transgender medical care to come out of the closet at my institution.

Thanks to the warrior women, I now know how to open the door.

